# Exploring the Impact of ACE Inhibition in Immunity and Disease

**DOI:** 10.1155/2022/9028969

**Published:** 2022-08-04

**Authors:** Delia Oosthuizen, Edward D. Sturrock

**Affiliations:** Division of Chemical and Systems Biology, Department of Integrative Biomedical Sciences, Institute of Infectious Disease and Molecular Medicine, University of Cape Town, South Africa

## Abstract

Angiotensin-converting enzyme (ACE) is a zinc-dependent dipeptidyl carboxypeptidase and is crucial in the renin-angiotensin-aldosterone system (RAAS) but also implicated in immune regulation. Intrinsic ACE has been detected in several immune cell populations, including macrophages and neutrophils, where its overexpression results in enhanced bactericidal and antitumour responses, independent of angiotensin II. With roles in antigen presentation and inflammation, the impact of ACE inhibitors must be explored to understand how ACE inhibition may impact our ability to clear infections or malignancy, particularly in the wake of the coronavirus (SARS-CoV2) pandemic and as antibiotic resistance grows. Patients using ACE inhibitors may be more at risk of postsurgical complications as ACE inhibition in human neutrophils results in decreased ROS and phagocytosis whilst angiotensin receptor blockers (ARBs) have no effect. In contrast, ACE is also elevated in certain autoimmune diseases such as rheumatoid arthritis and lupus, and its inhibition benefits patient outcome where inflammatory immune cells are overactive. Although the ACE autoimmune landscape is changing, some studies have conflicting results and require further input. This review seeks to highlight the need for further research covering ACE inhibitor therapeutics and their potential role in improving autoimmune conditions, cancer, or how they may contribute to immunocompromise during infection and neurodegenerative diseases. Understanding ACE inhibition in immune cells is a developing field that will alter how ACE inhibitors are designed in future and aid in developing therapeutic interventions.

## 1. Introduction

**Figure 1:**
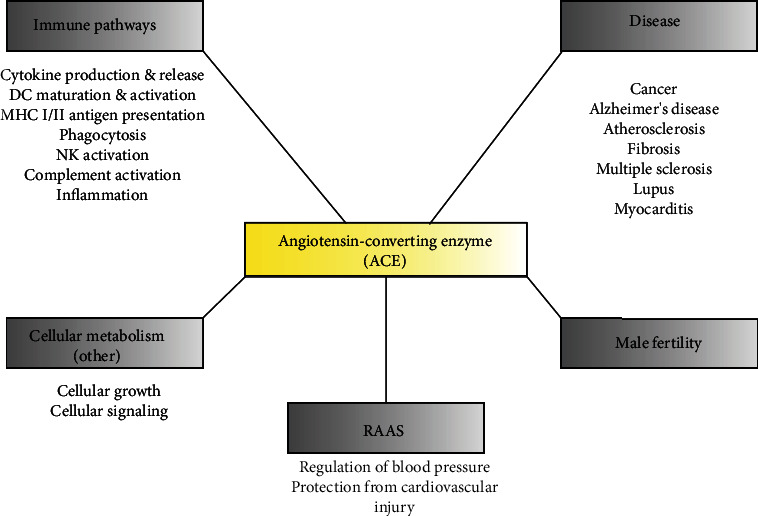
Physiological functions of angiotensin-converting enzyme (ACE). A summary of known or observed functions related to ACE activity in both human and murine models. ACE has been associated with several immune pathways related to clearing infection, primarily observed in murine models with high levels of baseline expression. In diseased states, including autoimmune conditions, ACE is elevated in the serum, but its overexpression also confers resistance to cancer and Alzheimer's disease progression. Local ACE may be involved in cellular growth and development, and ACE inhibition blocks the signalling cascades of important pathways. A lack of ACE results in decreased male fertility in mice. The main function of ACE in the RAAS is the regulation of blood pressure through Ang II-dependent actions, as either a vasopressor or vasodilator.

Angiotensin-converting enzyme (ACE) is well-known as a key regulator and component of the renin-angiotensin-aldosterone system (RAAS), which controls cardiovascular health, particularly blood pressure [1–3]. In hypertension, ACE is upregulated and active in converting its main substrate, angiotensin I (Ang I), to angiotensin II (Ang II). ACE inhibitors (ACEi) and angiotensin receptor blockers (ARBs) have been developed to treat hypertension and maintain cardiovascular health [4–6]. However, research has shown that ACE is involved in multiple aspects of human health, particularly the immune system due to its highly variable substrate cleavage (Figure 1) [7–11]. Much evidence has been published in the past decade that has contributed to a novel understanding of ACE as part of the immune system [7, 8, 11–13]. Although the precise mechanisms by which ACE exerts immune effect remain unknown, it is clear that ACE inhibitors can alter the response to immune challenge. Side effects regarding immune preservation have been poorly explored and much of this work requires expansion, particularly in humans. The present review is aimed at exploring the impact of ACE inhibition in light of the emerging role of ACE in key immune cell populations. By critically assessing the body of literature, we hope to identify areas that require more in-depth research and guide future studies to further our understanding of the impact of ACE inhibition.

## 2. An Overview of ACE in Immunity

Within the immune system, ACE exerts both beneficial and detrimental effects, particularly in fibrotic or atherosclerotic diseased states [4, 14]. These abilities are often due to Ang II-dependent signal transduction, but ACE is also directly involved in some immune signalling [7]. This section will briefly outline both the Ang II-dependent and Ang II-independent immune regulating abilities of ACE before exploring how these are affected by ACE inhibitors.

### 2.1. Ang II-Dependent Effects

The benefits and disadvantages to the immune system caused by Ang II have been thoroughly investigated. Ang II encourages proinflammatory responses and macrophage activation *via* the AT_1_ receptor (AT_1_R) [12]. When the AT_2_ receptor (AT_2_R) is bound by Ang II, anti-inflammatory and tissue repair responses are favoured by activated myeloid cells [12].

Inflammation is composed of three stages and is influenced by Ang II through AT_1_ and AT_2_ receptors [5, 15, 16]. In vascular permeability, prostaglandin and vascular endothelial cell growth factor (VEGF) production is stimulated *via* Ang II, thus controlling microvascular permeability as modulated through AT_1_R *via* cytoskeletal rearrangement [5, 15, 16]. Leukocytes, such as neutrophils and macrophages, are recruited, and Ang II upregulates the expression of E-selectins, VCAM-1 and ICAM-1, responsible for leukocyte adhesion and diapedesis into target tissues [15–18]. E- and P-selectin expression on endothelial cells is directly increased by Ang II and mediated through reactive superoxide (ROS) generation and AT_1_R [16]. Independent of blood pressure, ICAM-1 and VCAM-1 expression is enhanced by Ang II and regulated through a signalling cascade of AT_1_R and the MAP kinase pathway [5, 16]. Along with leukocyte migration, Ang II induces cytokine expression, including monocytic chemotactic protein (MCP-1), IL-8, and IL-18, which are involved in macrophage recruitment to the vascular walls [15]. Ang II also promotes increased production of cytokines by macrophages, including TGF-*β*, IL-1, IFN, and TNF-*α*, favouring monocyte differentiation and polarization thus enhancing phagocytosis directly [12, 16, 19]. During the recovery stage, Ang II may either exert profibrotic or antifibrotic effects, depending on whether it is bound to AT_1_R or AT_2_R [5]. Ang II induces fibrosis *via* the TGF-*β*-dependent and TGF-*β*-independent Smad-signalling pathways, where it induces abnormal vascular repair due to its influence on extracellular matrix deposition, growth factor, and collagen deposition within the vascular walls [15]. The antifibrotic action of AT_2_ receptors occurs *via* the blockade of the AT_1_ receptor and subsequent upregulation of AT_2_R, resulting in the inhibition of vascular inflammation, and favouring recovery [15].

Linking the adaptive and innate immune systems are the dendritic cells (DCs), which are stimulated to migrate, present antigens, and mature faster by exposure to Ang II [17, 20]. However, the proliferation and phagocytic activity of DCs is suppressed by Ang II [20]. The stimulation of DCs by Ang II upregulates DC-mediated T lymphocyte activation through the activation of the p65, NF-*κ*B, ERK1/2, and STAT1 pathways [20].

**Figure 2:**
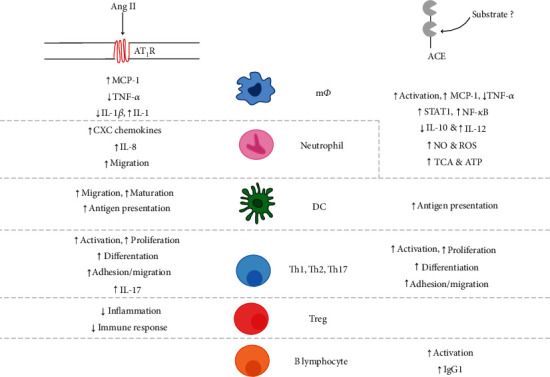
The roles of Ang II and ACE in innate and adaptive immunity. These functions are mediated through the AT_1_ receptor signalling cascade in different immune cell lineages where Ang II is present, but through unknown substrates or signals when ACE is utilised. Notably, most immune-related changes are observed in macrophages and neutrophils with respect to ACE, and there is minimal research regarding adaptive immunity. Abbreviations: m*Φ*: macrophage, DC: dendritic cell, Th-1,2,17: T helper lymphocytes, Treg: T regulatory lymphocyte.

In adaptive immunity, T lymphocytes are the main targets of Ang II modulation (Figure 2), particularly CD4^+^ T lymphocytes. Chronic Ang II presence induces the expression of early activation markers, including CD69, CD44, and CCR5, both *in vivo* and *in vitro* [17]. Furthermore, Ang II favours Th1 and Th17 proliferation, as locally produced Ang II stimulates increased IFN-*γ* and IL-17 and decreased IL-4 production, along with proliferation and differentiation of T lymphocytes [17, 21]. Transfer of T regulatory (T_regs_) lymphocytes in chronic Ang II-infused mice can prevent macrophage and T lymphocyte tissue infiltration, demonstrating that Ang II may influence T_regs_ and attenuate the inflammatory process [17, 21].

AT_1_R also has a protective role when expressed on myeloid cells and T lymphocytes [10]. In mice lacking AT_1_R, Th1 differentiation and the expression of proinflammatory cytokines including IFN-*γ* and TNF-*α* are increased. However, when AT_1_R is present, Th1 differentiation and proinflammatory cytokine release is suppressed [10]. In macrophages, suppressed M1 polarization is also seen with AT_1_R activation and reduces TNF and IL-1*β* levels [10]. The exact pathways by which AT_1_R modulates the myeloid and lymphoid proinflammatory populations are under investigation and may enable the reversal or prevention of hypertensive and renal fibrotic deterioration [10]. The potent modulating effects of Ang II over both the innate and adaptive immune systems is summarised in Figure 2 [17].

#### 2.1.1. Ang II-Independent Effects

The Ang II-independent immune response has been investigated to a lesser extent than the Ang II-dependent response, with unexpected but important findings [12, 17]. Fully understanding the role of ACE in immune modulation is complicated by its ability to recognise and cleave peptides other than Ang I. Semis et al. [22] established crucial evidence that ACE has many different peptide substrates using a discovery mass spectrometry-based analysis of mouse plasma [22]. These novel peptides ranged from approximately 4 to 30 amino acids in length. The biologically active C3 and C3f complement proteins are included as ACE substrates but, their activities after cleavage have not been described [22]. Earlier studies also identified the substrate promiscuity of ACE and observed Ang II-independent immune effects [10].

**Figure 3:**
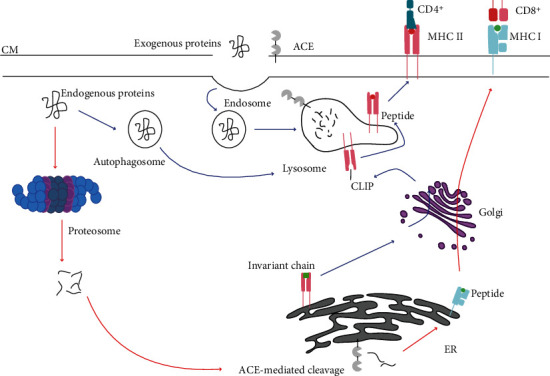
Schematic of the ACE-mediated cleavage of peptides for MHC Class I and II presentation. During MHC Class I (red arrows) antigen preparation, cytoplasmic proteins are processed into peptide fragments *via* the proteasome. These peptides can be further processed in the endoplasmic reticulum (ER) *via* ACE, which provides further alterations for increased specificity and selection by CD8^+^ T lymphocytes. The MHC Class II pathway (blue arrows) is for exogenous and endogenous protein digestion within endosomes and lysosomes. Newly synthesised MHC II associates with the invariant chain within the ER, before shuttling the complex to the endocytic pathway. The invariant chain is trimmed to CLIP which remains bound to MHC Class II. Once in the lysosome, CLIP binds HLA-DM to facilitate peptide binding to MHC Class II. The complex is then directed to the cell surface for antigen presentation to CD4^+^ T lymphocytes.

One such immune cell-specific action is the role that ACE exerts upon T lymphocytes regarding antigen presentation [23, 24]. MHC Class I peptide preparation is summarised in Figure 3, where ACE provides further cleavage of peptides through its carboxypeptidase activity [10]. Functional ACE is expressed in the endoplasmic reticulum (ER) of antigen-presenting cells (APCs), namely macrophages and dendritic cells, where it digests sample peptides and alters the MHC Class I peptide repertoire. However, these conditions were initially thought to require ACE overexpression [23]. Shen et al. [25] subsequently showed that ACE was capable of editing the C-termini of these intracellular peptides under physiologic conditions [25, 26]. Within mice, researchers have noted that other carboxypeptidases cannot correct a lack of ACE during MHC peptide digestion and presentation [26]. These alterations by ACE contribute to the large peptide repertoire required for the specialised adaptive immune response to stimuli [10, 12, 26]. ACE has also been implicated in altering the MHC Class II repertoire, the pathway of which is shown in Figure 3. As with MHC Class I, ACE can increase or decrease the presentation of certain types of peptides depending on how well it binds [24]. Zhao et al. [24] gave the first evidence of ACE involvement in the MHC Class II endosomal/lysosomal pathway, in both ACE-overexpressing (ACE 10/10) and wild-type (WT) mice [24]. Although several Ang II-independent immune alterations have been observed, the vast majority have been in murine models expressing high levels of ACE, discussed below.

#### 2.1.2. ACE Overexpression and Enhanced Immunity

ACE overexpression and its role in immunity have been highlighted in numerous reviews by Bernstein et al. [12, 19]. Animal models with ACE overexpression in immune cells have shown remarkable resistance to immunological challenge, including B16 melanoma. One such model is the ACE 10/10 model, designed to overexpress ACE 16 to 25-fold in murine macrophages in comparison to wild-type murine macrophages [12, 13, 25]. Macrophages have a variety of phenotypes and a large degree of plasticity due to their microenvironments and variable responses to cytokine secretion [27]. Macrophage phenotypes can be simplified into classically activated M1 (proinflammatory) and alternatively activated M2 (anti-inflammatory) phenotypes in response to the secretion of two cytokines, IFN-*γ* and IL-4, respectively [27, 28]. Classically activated macrophages (M1) are seen as antimicrobial and integral to the innate immune response, whilst alternatively activated macrophages are associated with host clean-up process and tissue repair [27–29].

The ACE 10/10 macrophages show enhanced immunity, elevated levels of IL-12 and nitric oxide (NO), and decreased levels of IL-10. These characteristics are associated with the pathogen- and tumour-killing ability of M1 macrophages which are linked to increased melanoma resistance in mice, partially due to the altered peptide processing pathways for MHC Class II molecules [24, 25]. ACE inhibitor treatment negates this effect entirely, unlike AT_1_R inhibition [25]. Since the enhanced tumour resistance continues with AT_1_R inhibition, it follows that this exaggerated macrophage phenotype is Ang II-independent and involves the ACE-mediated hydrolysis of peptides yet to be identified. The extent of ACE overexpression involvement in antigen presentation is still dependent on the amino acid sequence of the peptides during processing and will favour certain sequences over others [12]. Thus, ACE overexpression will not always increase the immunogenicity of peptides, and the enhanced immune response seen in ACE-overexpressing macrophages likely involves several mechanisms [12].

Another important disease state that has been studied in conjunction with ACE overexpression is Alzheimer's disease (AD) [30]. Macrophages recruited to the brain have increased ability to cleave *β*-amyloid plaques (A*β*), the pathogenic peptides associated with AD. Peripheral monocytes and macrophages then regulate neuroinflammation unlike the resident brain microglia [30]. Macrophages with ACE overexpression have an increased ability to maintain cognitive stability, memory, and function, unlike WT mouse macrophages. The ACE^10^ murine model developed by Koronyo-Hamaoui et al. [30] had lifelong ACE overexpression, but the administration of bone marrow-derived overexpressing monocytes and macrophages resulted in a similar enhanced protection of neurocognitive function in AD mice [30]. ACE 10/10 monocytes and macrophages were recruited to the brain and were effective against both soluble and insoluble forms of the amyloid *β* (1-42) peptides, thus preserving the cognitive performance of the mice. These results suggest that blood enrichment with ACE-overexpressing monocytes and macrophages may be an effective and promising treatment for AD in human patients. Additionally, ACE has been identified as a candidate significant risk gene through integrated genome- and proteome-wide association studies (GWAS and PWAS), supporting a previous GWAS meta-analysis of 94 437 late-onset Alzheimer's patients wherein ACE was identified as a candidate AD risk factor gene [31–33]. Individuals with a single-nucleotide polymorphism (SNP) in ACE have a 45-fold increased risk of developing AD compared to those without it [30, 34, 35]. The single-nucleotide variation (SNV), rs4277405, ACE allele increases AD risk as a homozygous insertion (II), whereas heterozygous (ID) or homozygous deletion (DD) alleles lower AD risk [32]. Individuals with the DD allele have increased ACE plasma levels and a lowered risk of developing AD [30, 36, 37].

The variable substrates of the C- and N-domains could be utilised for the induction of ACE-mediated enhanced immunity if one domain is more active in this role than the other, or for controlling autoimmune states where ACE is implicated. Using intradermally injected B16 melanoma cells, in the ACE 10/10 murine model, it was determined that melanoma resistance requires an active C-domain, whilst a missing or inactive N-domain had no effect on tumour growth [38]. This effect is independent of Ang II, bradykinin, and substance P, according to experiments using receptor inhibitors and transgenic mice with no ability to produce angiotensin peptides [23, 25, 39]. This view highlights that a separate ACE substrate and product of unknown origin is responsible for the enhanced immune response compared to WT and is mediated through ACE C-domain catalytic activity [22, 38].

The increased catalytic activity of the ACE C-domain in macrophages drives their proinflammatory state over myeloid suppressor cell development [38, 40]. Myeloid-derived suppressor cells (MDSCs) suppress several aspects of the immune response but also act in several pathologies including cancer, sepsis, and chronic inflammation. MDSCs are composed of myeloid progenitor cells including macrophages, granulocytes, and dendritic cells [40]. In murine models lacking ACE, increased populations of immature myeloid precursors are observed, whilst ACE overexpression results in decreased MDSC populations and increased proinflammatory macrophages [40]. Through the different crosstalk pathways of macrophages, the net effect of the changes observed in cytokine production is an increase in the immune response compared to in WT mice [38]. This enhanced immunity continues with bone marrow-derived macrophages transplanted into WT mice or with lipopolysaccharide (LPS) challenge to bone marrow-derived macrophages in culture [12, 25]. ACE 10/10 mice also have an enhanced adaptive immunity, mediated through antigen presentation from the macrophages to CD8^+^ T lymphocytes that are required for efficient tumour-specific immune attacks [25]. As CD4^+^ T lymphocytes act in MHC class II antigen presentation, the mechanism by which B lymphocytes react to ACE overexpression is also important as B cells require CD4^+^ activation [12, 24]. Humoral adaptive immunity is also enhanced, as noted when B lymphocytes produce more anti-OVA (ovalbumin) antibodies in ACE-overexpressing mice compared to in WT mice [24]. In particular, the IgG1 antibody was up to 20-fold higher in ACE-overexpressing mice than in WT mice and is an important “first-responder” antibody during infection [12].

Enhanced innate immunity has been observed in the ACE 10/10 model following challenge with *Listeria monocytogenes* and methicillin-resistant *Staphylococcus aureus* (MRSA) [41]. Resistance to bacterial growth is particularly evident in MRSA-infected skin lesions of ACE 10/10 mice. However, ACE catalytic activity is not directly responsible for bacterial killing but instead primes macrophages, requiring IFN-*γ* to elicit the enhanced immune response. As ACE is involved in peptide processing for antigen presentation, the critical role of ACE overexpression may be enhanced peptide presentation and immune pathway activation, rather than the creation of a separate microenvironment [12, 41]. The enhanced innate immunity of the ACE 10/10 mouse model is also due to heightened iNOS and nitrogen intermediate expression, combined with increased ROS production. This observation was strengthened by using an iNOS inhibitor which abrogated the increased resistance to bacterial infection in these mice compared to in WT mice [41]. These observations coincide with the iNOS- and NADPH-related mechanisms of increased NO or ROS production as the driver in macrophage ACE-mediated vascular inflammation [41, 42].

When ACE is overexpressed in neutrophils, their bactericidal and oxidative responses are enhanced (Figure 2) [43]. Under physiological conditions, ACE expression increases in activated neutrophils challenged with MRSA. Within the NeuACE model, neutrophils then increased their basal ACE expression to between 12- and 18-fold higher compared to unchallenged neutrophils. NeuACE neutrophils exercise greater bactericidal ability *via* superoxide production (phagocytosis included) and neutrophil extracellular fibre (NET) formation, emphasising a direct relationship between bacterial killing and ACE production [12, 43–45]. If ROS production is inhibited by NADPH oxidase inhibitors or ACE inhibitors, the enhanced immune effect is lost in NeuACE neutrophil and ACE 10/10 macrophage models [12].

#### 2.1.3. Proposed Mechanisms and Metabolic Changes

Studies regarding the mechanisms and pathways behind the enhanced immune response from ACE overexpression are still in development. One such study by Cao et al. [44] observed increased cellular oxidative metabolism and ATP production in ACE 10/10 macrophages and NeuACE neutrophils [44]. ACE-overexpressing macrophages and neutrophils have an increase in TCA cycle intermediates which in other immune cells is used to compensate for a burst in glycolysis and signalling molecules [29, 44, 46]. Increased TCA cycle intermediates also suggest that the macrophages and neutrophils may be using these as precursor molecules for other bactericidal products aside from superoxide production to enhance their phagocytic ability [29, 44, 46].

Identifying the substrate responsible and the intracellular changes that facilitate this enhanced immune phenotype in ACE-overexpressing macrophages would be important advances in ACE therapeutic manipulation. Currently, the peptide(s) responsible for the enhanced immune effects is unknown with a new library of potential substrates identified by Semis et al. [22, 47]. In addition, several of the peptides identified by Semis et al. [22] have also been identified in human plasma. This observation suggests that the novel substrate or product responsible for activating the alternative ACE pathway in enhanced immunity is likely present in both humans and mice.

ACE-overexpressing murine models have been used to study these enhanced immune responses to disease challenges whilst little work has focused on human ACE-overexpressing cell lines. This may limit the potential of ACE-overexpression therapies in humans, but in-depth cellular proteomic studies may lead to explanations of how to utilise these differences whilst also aiding our understanding of the impact of ACE inhibition or loss on immunometabolism.

## 3. ARB and ACEi: Effects on Immune Abilities

As our need for new immunotherapies grows, the effects of common medications such as antihypertensives need to be better understood. Human and animal studies are crucial to our understanding and management of hypertensive patients with underlying conditions or complications that may be exacerbated by medications, particularly immunocompromised individuals with increased susceptibility to infection. In human neutrophils, some ACE inhibitors and ARBs are able to negatively affect cellular antimicrobial responses particularly extracellular and intracellular clearing of bacterial invaders [48]. In other immune cells, very little is known of how well they take up these drugs, and if their phagocytic or bactericidal abilities are impacted by them.

Angiotensin type I and II receptors are critical in the proinflammatory and reparatory pathways associated with Ang II production. When bound by Ang II, these receptors regulate signal transduction throughout the body to mediate cellular recruitment and actions [5]. This role of ACE and Ang II is mostly beneficial but may also cause vascular injury such as fibrosis and atherosclerosis when overactive. Thus, careful mediation of Ang II effects through its receptors is required. ARBs act on these pathways through the prevention of Ang II binding to the relevant receptor, with the most common ARBs preventing Ang II type I receptor (AT_1_R) binding, in turn preventing cytokine and chemokine secretion, leukocyte recruitment, and extravasation of immune cells to the site of inflammation (Figure 2). ARBs that are frequently prescribed include losartan, candesartan, valsartan, telmisartan, and olmesartan [49]. Although substantial work has been performed on ARBs and Ang II, these inhibitors are not the main focus of this review and will be briefly discussed in conjunction with ACE inhibitors.

Little information is available on inhibitor uptake within tissue and cell culture, and the data available is often inconsistent in terms of mechanisms and intracellular levels determined [50]. Detailed knowledge of the intracellular uptake of ACEi is important for drug safety and pharmacokinetics outside of serum levels. Furthermore, intracellular ACE may be useful in determining off-target effects of candidate compounds, and where gene knockout is either unfavourable or not possible. ACE inhibitors targeting a particular domain are being developed to improve unwanted side effects and increase drug specificity for targeted disease treatment [6]. The most common prescription ACEi are captopril and ramipril in the US and EU, respectively [51]. Common ACE inhibitors include enalapril, fosinopril, captopril, and lisinopril. Since its introduction in 1978, captopril has been extensively used for investigating ACE inhibitors and understanding their mechanisms and long-term effects. Captopril has effective antihypertensive action without a known mechanism in both human and animal studies [52]. The mechanisms by which captopril interferes with other systems and conditions have been studied in detail, and it has been suggested as an immunotherapeutic candidate. Lisinopril is another common ACE inhibitor but, like other clinical ACEi, is associated with off-target effects due to inhibition of both the N- and C-domains. To counteract this, lisinopril-tryptophan (Lis-Trp) was developed as a C-domain-specific inhibitor that would only target the conversion of Ang I into Ang II, whilst preserving bradykinin metabolism [53, 54]. Although lisinopril has been extensively studied, its counterpart Lis-Trp has not been approved for use in humans.

ACE inhibitors in immunity are usually studied in the context of autoimmune disorders, often regarding a beneficial patient outcome, but with a lack of research on the cellular uptake of ACEi, or which processes are impacted. Instead, these studies measure proinflammatory cytokines and markers that have been connected to the disease of interest. Further research regarding their impact on macrophages and other immune cells is required and could improve patient outcomes, particularly in a nosocomial setting, where the number of drug-resistant infections is rising, and also in the wake of the ongoing coronavirus (SARS-CoV2) pandemic.

Captopril and lisinopril investigations within an immune scope have shown that ACE may exert an important regulatory control over several pathways, but these data are often contradictory, emphasising the complexity of both ACE functionality and the immune response. Although ACE has a wide range of substrates, it often favours one domain over the other for a particular molecule [8, 55]. For instance, Ang I is the preferred substrate of the C-domain but can also be cleaved by the N-domain. The main purpose of ACEi has been the treatment and maintenance of cardiovascular conditions, including heart failure and hypertension. However, ACE is involved in several immune and biological pathways either through systemic or local RAAS systems. Thus, ACE may also serve as a useful target for the treatment of inflammatory conditions or autoimmune diseases since all immune cells express local RAAS components [7, 8, 12, 56].

Generally, a reduction in key inflammatory cytokines has been observed with ACE inhibition [57–60]. Several studies have concluded that ACEi are beneficial for preventing renal failure, proteinuria, and the amelioration of the inflammatory response within autoimmune or cardiovascular diseases [49, 61–64]. However, few studies have considered the effects of the inhibitors on immune pathways in otherwise healthy individuals, and how these processes, including peptide processing and inflammation, may be altered [48].

### 3.1. ACE Inhibition in Autoimmunity and Inflammation

Autoimmune studies on ACEi have shown mixed evidence of improved patient outcomes, and larger studies are required to determine the overall benefits. Furthermore, limitations of these studies are often that they measure cytokines only, rather than drug uptake, markers, cell function, and cell number. These treatments would be additive to our current knowledgebase but would not be adequate replacements of current autoimmune therapies, and more in-depth analysis is required to understand the role of ACE in these conditions.

ACE and Ang II have been studied in relation to vascular inflammation and injury where their inhibition results in reduced inflammation, leukocyte recruitment, and injury to the vascular walls, which are key characteristics of chronic inflammation and autoimmune conditions [10]. ACE inhibitor treatment has not been associated with autoimmunity in humans to date [65]. However, this treatment has improved the outcome of some autoimmune conditions, such as rheumatoid arthritis (RA), multiple sclerosis (MS), and lupus, by decreasing proinflammatory cytokine production. Cytokines such as IL-12 and TNF-*α* are significantly reduced in autoimmune disease models receiving ACEi, and in conjunction with AT_1_R blockers, autoantibodies, cytokine expression, and immune cell activation are also decreased [17, 21, 65].

#### 3.1.1. Multiple Sclerosis and Lupus

Individually, ARB treatment of MS models in mice, otherwise known as experimental autoimmune encephalomyelitis (EAE) and experimental autoimmune myocarditis, has also resulted in improvements to outcomes [57, 66]. ACE is elevated in MS patients and EAE murine models and is involved in T cell activation through antigen presentation [23, 24, 39, 64]. In EAE, characterised by high levels of Th1/Th17 lymphocytes, lisinopril-treated mice have increased T_reg_ cell activation and decreased IL-12, which is an important T helper cell proliferation and maturation activator [57]. Given lowered T helper cell populations in these mice, lisinopril may improve EAE outcomes by reducing inflammatory signalling and cytokine production by these cell types. Captopril also reduced IFN-*γ* production and activated SOCS-1 and 3, suppressors of proinflammatory cytokines in EAE [64]. Captopril was able to protect against further brain and spinal cord inflammation in a Lewis rat model with no leukocytopenia development. These protective functions are thought to involve T lymphocytes that are not involved in antigen presentation, such as memory T cells and T_reg_ cells [64].

A study on the effect of protease inhibitors on murine T lymphocytes revealed that lisinopril was able to inhibit ACE activity at 1 *μ*M whilst captopril required higher concentrations. However, captopril forms disulfide bonds quickly, and its true inhibitory nature is difficult to measure without excluding oxygen [67]. Other serine proteases may also be influential in T lymphocyte ACE activity, since leupeptin was able to inhibit ACE and aminopeptidase B inhibitor, bestatin, treatment increases ACE activity [67]. Thus, ACE inhibition may affect T lymphocyte activation and proliferation by altering the immune response through a change in cell surface signals, although the full mechanism remains unknown [67]. Lisinopril treatment also reduces TGF-*β*1 production and improves the symptoms of murine thyroid granulomatous experimental thyroiditis disease by reducing the proinflammatory cytokine profile and collagen deposition [68]. Although stronger immunomodulation was seen using the ACEi, lisinopril, the ARB, candesartan, could decrease proinflammatory cytokine production and increase immune modulating messengers, such as TGF-*β* and IL-10, significantly [57]. However, larger ACEi studies are required to fully grasp their role in ameliorating autoimmune disorders such as lupus and EAE [57].

**Figure 4:**
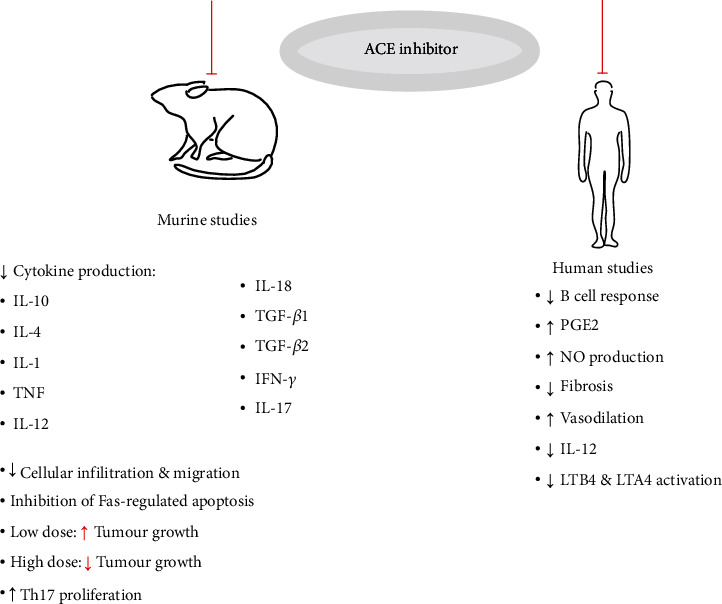
A summary of immune cellular and cytokine changes with ACE inhibitor administration. Importantly, ACEi treatment results in a significant reduction in proinflammatory cytokine production, but these changes appear to be dose-, host-, and disease-dependent. Captopril and lisinopril have been extensively studied in both mice and human autoimmune models but the mechanisms of action are not well understood.

ACE polymorphisms have also been associated with severe systemic lupus erythematosus (SLE) and ACE inhibitors slow SLE progression. SLE patients have increased ACE serum levels, hypothesized to result from ACE polymorphisms similar to Alzheimer's patients [58]. The ACE ID allele has been identified in 644 SLE families and 39 SLE patients in comparison to 79 controls through genomic screening [69, 70]. The 644 SLE patients and their family members were genotyped for three ACE gene polymorphisms, an Alu insertion/deletion (ID), 23949 (CT)_2/3_, and 10698 (G)_3/4_ [70]. Rabbani et al. [69] screened ACE ID and 2350 G > A dimorphisms, finding only the AA allele to be associated with severe SLE in humans. Untreated SLE progresses by depositing immunocomplexes within the kidneys and autoantibody production that results in kidney disease. Captopril has been administered in a lupus nephritis mouse model resulting in reduced levels of Th2-related cytokines, TGF-*β*1 and TGF-*β*2 [62]. With its noted immunomodulatory effects in several diseases, including EAE, RA, and schistosomiasis, Odaka and Mizuochi [71] administered captopril to murine T cell hybridomas and observed the inhibition of Fas-regulated apoptosis [64, 71, 72]. Inhibition of apoptosis is prevented through the captopril sulfhydryl redox reaction which prevents its signalling cascade [71, 73]. Captopril also preserves cognitive function as it passes into the brain and prevents microglial activation [74, 75]. Interestingly, captopril can also induce SLE, and its inhibition of activation-induced T lymphocyte apoptosis may play a role in activating the observed autoimmune response [71]. The administration of captopril to active SLE mice results in a decrease in spleen IL-4 and IL-10 production, but no reduction in autoantibodies. Captopril is therefore able to reduce renal lesions and reverse kidney disease, but the mechanisms remain unclear [62]. Lupus-prone mice also had decreased cytokine production with captopril treatment (Figure 4), possibly *via* a novel IFN-*γ* and ACEi pathway, which subsequently decreased IFN-*γ*.

In MRL/*lpr* mice, glomerular damage and renal chemokines are reduced with ACEi treatment. Clinically, the LUMINA cohort observed that ACEi therapy reduced SLE disease activity and prevented renal involvement [76]. Fosinopril is able to inhibit STAT3 activation, in mice, which is important in various cytokine signalling cascades. STAT3 also plays a role in cellular growth, inflammation, and embryonic development [77]. Enalapril is able to modulate the inflammatory response through increasing IL-10 and increasing splenic CD4^+^ T lymphocyte activation and migration, which disagrees with the observations of De Albuquerque et al. in 2004 where IL-10 and TGF-*β* were decreased after captopril treatment of lupus-prone mice [62, 72]. Enalapril resulted in an increase in CD4^+^ T lymphocyte maturation and IL-10 production, with macrophage polarization shifted toward the M1 state. IgG1 production was unaffected by enalapril treatment; however, IgG2c levels were increased after 4 weeks in lupus-prone mice [78]. Whether these changes are Ang II-dependent or Ang II-independent is unknown and should be investigated further in the future by treating with ARBs in parallel. Information regarding autoantibody production is limited and contradictory since most studies concentrate on T lymphocyte populations, and not B lymphocytes or innate immune cells such as macrophages or neutrophils. Further supporting the application of captopril as an immunotherapeutic, lupus-induced neuroinflammation, and immunocomplex deposition within the brain and kidneys decreased and early disease onset was reversed after short-term captopril treatment [75]. Lupus patients are now prescribed ACEi with immunosuppressants, indicating the importance of ACE in SLE progression and the benefit observed in those on ACEi over ARBs or no antihypertensive medications [76, 79]. In particular, captopril may be of use in other autoimmune and inflammatory areas as described for AD and RA.

#### 3.1.2. Alzheimer's Disease and Rheumatoid Arthritis

Since captopril can cross the blood-brain barrier and reduces neuroinflammation in lupus, further studies in Alzheimer's models are warranted [75]. Many elderly patients use ACE inhibitors to treat hypertension and heart failure. Although considered safe, some studies have suggested using ARBs over ACEi since ACE is capable of cleaving *β* (1-42) and reduced ACE levels are associated with increased AD risk [36, 80]. Given that ACE overexpression maintains murine cognitive ability by increased A*β* degradation, ACE inhibition may be detrimental to patients at higher risk of AD development including those with the II allele [30, 81]. The novel ARB and neprilysin (NEP) inhibitor, Entresto (a co-crystal of sacubitril and valsartan), has been approved for use in elderly patients to treat heart failure. However, the effects of this ARB/NEP hybrid should be investigated with regards to Alzheimer's progression, as NEP inhibition or loss can increase A*β* accumulation and enhance mitotic protein activity, since NEP is important for A*β* degradation in the brain [82, 83]. Comparing ACEi and ARBs in AD patients negative for APOE*ε*4, ACEi use was associated with increased memory loss and decreased attention preservation when compared to ARBs. Thus, reduced ACE is associated with higher risk of AD development and progression, supporting the use of ARBs over ACEi in elderly patients [31–33].

Since captopril is also able to reduce renal symptoms in lupus models, it may also improve RA symptoms [84]. A small study observed improved RA symptoms in 66% of patients receiving captopril; however, this cohort consisted of only 15 individuals, and a larger sample group should be studied in future [17, 85, 86]. Additionally, the nonthiol ACEi pentopril had no effect on RA patients, and the improved RA symptoms observed after captopril administration were attributed to its thiol group, similar in structure to the immunosuppressant penicillamine [85, 87, 88]. ACEi and ARBs do not significantly improve inflammatory RA symptoms, despite decreased C-reactive protein (CRP) levels [87, 88]. Healthy PBMC cultures receiving captopril had decreased B cell responses that were monocyte-dependent and prostaglandin-independent (PGE2) [84, 89, 90]. Furthermore, Ang II-mediated PGE2 production suppressed T lymphocyte proliferation in the presence of ACE inhibitors, both *in vitro* and *ex vivo*. However, in combination with indomethacin and ACEi, PGE2 production is immunostimulatory and promotes antibody-mediated T lymphocyte proliferation [84]. Nitric oxide and PGE2 production were also increased with captopril treatment, which prevented monocyte/macrophage activation, and may explain benefits observed in atherosclerotic patients [17, 84–86].

#### 3.1.3. Atherosclerosis and Vascular Inflammation

During inhibition of NF-*κ*B, Marchesi et al. [15] reported reduced expression of IL-6, VCAM-1, and MCP-1, and ACE inhibitor treatment also reduced NF-*κ*B activity, thereby preventing vascular inflammatory stimulation that is associated with atherosclerosis [15]. ACE inhibition reduces circulating and urinary inflammatory cytokine markers, further supporting its role in mediating the immune response. The inhibition of ACE results in reduced damage within a cardiovascular and renal context, but its expression in immune cells specifically results in an altered immune response and cellular actions [10].

*In vitro* studies have shown that ACE inhibitor treatment diminishes cell proliferation of myeloid progenitor cells [16]. Furthermore, ACE inhibition in ApoE-deficient mice resulted in a decrease in adhesive molecules and chemoattractant expression in myeloid cells that was also associated with reduced oxidative stress and eNOS production [49]. In atherosclerosis, immunocomplexes form between ApoB, LDL, and antibodies IgG and IgM. When infused with Ang II, immune cells accumulate within the vascular walls, suggesting an inflammatory response which can be ameliorated using ACEi. A 12-week perindopril treatment resulted in decreased immune complex deposits in an atherosclerotic model [91]. These improvements lead to increased endothelial integrity due to autoantibodies produced against ApoB-derived peptides, as well as increased B cell activation, which prevents complex build-up [91]. When RAAS inhibitors are administered in murine models, including those for AT_1_R and ACE, the proinflammatory effects of Ang II are prevented, and there is decreased MCP-1 produced in atherosclerotic lesions [15]. RAAS blockade decreases monocytic infiltration into lesions and aortic tissues, along with MCP-1 and CD11b attenuation in circulating monocytes, benefitting patient outcome and improving health [16]. In atherosclerosis, diagnosis may be independent of Ang II and AT_1_R activation by means of the CCR9-CCL25 axis, which aids in recruiting macrophages for plaque formation [92]. When captopril and other ACEi are administered, cellular infiltration decreases, and the axis is mediated to prevent proinflammatory cytokine production [92].

Long-term ACEi usage also results in undesirable BK build-up which causes vascular injury and increased endothelial permeability or angioedema. Joint NEP and ACE inhibitors, such as omapatrilat, have been designed to block ACE-dependent Ang II production and decrease NEP-dependent degradation of vasodilators [93]. However, these can have more serious side effects than ACE inhibitors alone when targeting both ACE domains, due to BK accumulation [93]. Combination therapies alongside domain-selective ACE inhibitors have been explored to reduce these side effects and maintain BP control. Recently, NEP and C-domain combination therapy has been investigated in mice and *ex vivo* in human plasma. Reduced BP and increased BK degradation were observed. These findings are clinically important for the ACEi lisinopril-tryptophan and NEP inhibitor sacubitril, where hypertension was normalised without the loss of endothelial function and vascular permeability [94]. Furthermore, joint NEP and ACE inhibitors could be beneficial if designed to favour ACE C-domain over N-domain binding [93]. In contrast, sacubitril ARB therapy had no significant improvement in hypertensive mice, whereas NEP and ACE inhibition using omapatrilat was able to reduce BP but did not prevent BK build-up in mice, preventing stabilisation of Ang II and BP levels. Immunologically, these combination therapies could control and prevent chronic inflammation and injury in patients, allowing for efficient hypertensive treatment and prevention of cardiac failure [83, 94].

The ACE inhibitor quinapril can improve proteinuria or prevent its onset through reducing TGF-*β*1 gene expression and reducing matrix protein production [61]. These changes were observed in a murine glomerular nephritis model under normotensive conditions. Overall, renal ACE appears to be more affected by ACEi when administered to treat certain diseases, such as nephritis and glomerulosclerosis. The cardiac protection that lisinopril provides is prevented by hyperlipidemia when heightened Ox-LDL and Ang II levels cause increased dendritic cell maturation and migration [95]. These cells are crucial for antigen presentation and T cell activation [23, 39]. Furthermore, these conditions promoted increased IL-6 and TNF and upregulated maturation markers such as CD86 and CD83. These markers are also common indicators of atherosclerosis and myocardial infarction, but lisinopril is able to protect against these when Ox-LDL remains low [95]. In contrast, ACE overexpression results in decreased atherosclerotic lesions, which is also observed with ACE inhibition and challenges the current understanding that myeloid ACE is required for atherosclerotic progression, since enhanced clearance of plaques was observed in ACE10/ApoE mice [96]. As ACEi are designed to treat CVD and cardiac failure, it follows that myocarditis should see effective control when subjected to these therapeutics.

#### 3.1.4. Myocarditis and Fibrosis

Myocarditis is an inflammatory heart condition with reduced macrophage infiltration and increased myocyte necrosis. In an experimental myocarditis model, significant improvements were observed upon captopril regimen initiation [97, 98]. Captopril could reduce inflammation through increasing free radical scavengers and lowering cell-mediated immunity, as evidenced through reduced myosin and ovalbumin delayed-type hypersensitivity [97, 98]. Prolonged ACEi treatment may cause Ang II-independent type IV hypersensitivity in patients that are predisposed, *via* ACE substrates such as SP and BK. However, these may be ACE inhibitor-dependent, requiring individual studies [99]. Captopril also acts by preventing Ang II production, halting AT_1_R signalling and benefiting several myocardial conditions or infections [97]. This mechanism was not shown to affect cell proliferation and cytokine levels in T lymphocytes, but rather their adhesion and motility [98]. The ARB telmisartan was able to ameliorate myocarditis symptoms and decreased oxidative stress and inflammatory cytokine production [66]. ARBs can control the proinflammatory effects of Ang II and the RAAS without impacting ACE production and the natriuretic pathways. These compounds act by preventing signal transduction after Ang II binds to AT_1_R, including blocking ROS, NO, or inflammasome activation through the NF-*κ*B or Ets-1 transcription factors [15]. Thus, ARBs may be favoured over ACEi to prevent hypersensitivity reactions whilst controlling inflammation.

Perindoprilat can prevent fibrosis by means of the bradykinin pathway, by regulating lipoprotein receptor (LPR) pathways and increasing tPA signalling, resulting in improved fibronectin breakdown, termed “ACE inhibitor-independent antifibrotic action” [100]. During chronic heart failure, ACE inhibitors are able to reduce inflammatory profiles, but patients still have elevated NF-*κ*B and AP-1 levels with minimal benefits. If administered during acute myocarditis, some patients may experience reduced NF-*κ*B levels, which regulates a host of innate immune pathways and inflammation [101]. Captopril has also benefitted systemic sclerosis patients by preventing fibrosis and allowing vasodilation to improve myocardial function when given as a long-term treatment [102]. Captopril treatment also reduces the levels of IL-12, a macrophage activation marker that aids in cell-mediated immunity and is upregulated in ACE 10/10 murine macrophages [103]. Both captopril and lisinopril can decrease IL-12 production in human PBMCs, which may explain the reduced levels in sera of autoimmune and cardiovascular patients [103]. Future studies should determine if any cell-mediated pathways are dysregulated by captopril or lisinopril, which may negatively impact microbial clearance ability in the human PBMCs as expected based on the ACE 10/10 murine model.

#### 3.1.5. Inflammatory Bowel Disease

A small inflammatory bowel disease (IBD) study in humans noted that ARBs were able to significantly reduce hospitalization, steroid use, and IBD surgeries in comparison to ACEi [104]. The effects of an imbalance in Ang II signalling on TGF-*β*, TNF-*α*, and fibrosis is thought to impact the severity of IBD [105, 106]. In a murine gastrointestinal inflammatory model, ACE shedding was observed *ex vivo*, alongside increased corticosterone levels, further implying that ACEi and ARBs may aid in controlling IBD [107]. Once AT_1_R is inhibited, leukocyte migration and adhesion within the gastrointestinal tract are also decreased due to MAdCAM-1 suppression, a key mucosal adhesion molecule associated with gastrointestinal inflammation [105, 108, 109]. Thus, ARB administration to IBD patients results in improved clinical outcomes within six months of treatment where ACE inhibition did not show significant improvements and may therefore serve as a new treatment plan for severe IBD cases [105].

### 3.2. ACE Inhibition in Cancer

The role of ACE in the tumour microenvironment and cancer has been the subject of intense research. ACE overexpression results in reduced tumour growth in a B16 melanoma murine model, but when ACE activity was inhibited or knocked out, tumour size reverted back to wild-type proportions [25]. Ang II signalling also partly controls tumour progression but no differences in new cancer occurrence have been reported for ACEi or ARBs [110]. Instead, ACEi and ARBs can be used to manage chemotherapy side effects. Retrospective studies note conflicting outcomes in several human meta-analyses, but overall, no heightened risk is observed [111, 112]. ARBs and ACEi may improve patient outcome in several cancers, including breast, colorectal, pancreatic, and gastric cancer. Currently, the exact antitumour mechanism associated with ACEi, and ARBs has not been elucidated. However, Ang II-dependent signalling is halted and thought to reset angiogenesis, inflammation, and cellular proliferation [110].

Mice receiving losartan experience later onset of mammary tumours compared to their untreated counterparts, supporting Ang II involvement [113]. In contrast, low doses of the ACEi, captopril, promote tumour growth by downregulating CD8^+^ T cell function in mice, whilst higher doses inhibit tumour growth [114, 115]. In T cell hybridomas, captopril was unable to slow tumour growth, and in other models, captopril induces SLE syndrome, causing autoimmunity by interfering with the self-tolerance mechanism [71]. Captopril, therefore, appears to have two mediation arms for cancer progression that are dose-dependent and may also be dependent on cancer type [25, 114]. During early cancer stages, captopril may be beneficial for inhibiting tumour growth by improving CD3^+^ T lymphocyte infiltration, but during late stages, captopril decreases lymphocyte infiltration and alters PD-1 checkpoint expression [56]. Long-term treatment is postulated to reduce the chance of cancer progression; however, these studies have only investigated immunocompromised individuals and nonimmunogenic cancers [114]. Within immunogenic cancers or tumour types, captopril enhanced tumour growth and decreased macrophage and T cell proliferation [114]. Colorectal cancer recurrence and liver metastasis were halted by captopril treatment in mice after undergoing partial hepatectomy. Using flow cytometry, captopril treatment reduced MDSCs and stimulated PD-1 expressing resident memory T cells, linked to effective cytotoxicity in some tumour microenvironments [116]. This suggests captopril may be an effective adjunct therapy for colorectal cancer patients, but it is not known if these antitumour effects are directly related to captopril reducing tumour burden or form part of its immunomodulation abilities [116].

Additionally, perindopril attenuates intestinal epithelial injury and preserves goblet cell function in rats [117]. During immunosuppression or cancer treatment with methotrexate (MTX), intestinal injury occurs, and the mucosal immune cell population induces inflammation and oxidative action. Perindopril administration in conjunction with MTX in rats downregulated the inflammatory TLR4/NF-*κ*B and c-Fos/c-Jun pathways, allowing cytoprotective PPAR-*γ* and SIRT1 signalling to prevent oxidative stress [117]. Thus, as with captopril, perindopril and other ACEi can contribute to controlling and preventing inflammatory injury in cancer patients. However, these benefits are likely Ang II-dependent despite ACE inhibition, thought to be *via* decreased Ang II, increased protective Ang 1-7 peptide, and the associated cellular modifications. No ARBs were studied alongside these treatments, limiting whether the same results could be obtained using ARBs without the buildup of potentially harmful ACE substrates. Whilst simultaneous chemotherapy and ACE inhibition may benefit patients, perioperative conditions and infection may detrimentally alter the immune cell population and protection.

### 3.3. ACE Inhibition during Infection

Microbial challenge occurs every day and usually does not require antimicrobial or medicinal intervention. However, as the incidence of antibiotic resistance increases, alternative treatments and known drug interactions with pathogens and our immune cells are of growing importance. Exploring how ACE inhibition impacts microbial clearance is only now coming to the forefront, but it is expected to be crucial in our understanding of ACEi safety, particularly in light of the ongoing coronavirus pandemic.

#### 3.3.1. Chagas Disease

Parasitic infections are a common occurrence in the developing world but are limited in scope with regards to ACE. In Chagas disease or *Trypanosoma* infection, ACEi decrease fibrosis and cardiovascular inflammation. ACE inhibitors are thought to prevent cardiac inflammation and inhibit parasite infiltration [118–121]. However, these benefits are negligible and cannot be substituted as treatment for either condition [119, 122]. Captopril and enalapril have both been investigated as cardioprotective drugs, and although parasite infiltration was increased, leukocyte infiltration into cardiac tissue was improved, with reduced inflammatory cytokine profiles observed after treatment [118, 119, 121]. Captopril also downregulates IL-10, thus inducing Th17 cell activation, and can potentiate macrophage infection and upregulate IL-17 production [120, 121]. The Th17 cell population has also been implicated in autoimmune conditions such as MS, and thus, captopril administration may have an immune-altering effect on patients resulting in aggravated inflammation, which may be genetically dependent on host ACE polymorphisms [120]. Lisinopril treatment has no benefit in *T. cruzi* infection and does not affect cytokine production pathways in coxsackie virus-induced myocarditis [121, 123]. Long-term studies are required in these experimental models, as acute infection may differ from chronic infection in terms of patient morbidity [121]. With ACE-associated parasitic infections being uncommon and mostly having effective treatments or preventative measures in place, a more pressing field of study is ACE in bacterial and viral infections.

#### 3.3.2. Bacterial Infections

During bacterial infection, ACE is integral in antigen presentation and in clearing the infection [23, 24]. Neutrophils treated with ramipril or lisinopril had a decreased ability to clear MRSA infection in a human pilot study, whilst ARBs had no significant effect [48]. The p38-MAPK pathway and leukotriene B4 (LTB_4_) activity have been highlighted as important factors in ROS, chemotaxis, and cytokine production in neutrophils.

Captopril treatment prevents neutrophil activation through a potential ACE-related pathway in LTB_4_ activation, an inflammatory signal molecule involved in many conditions, such as psoriasis and atherosclerosis [48, 124]. LTB_4_ acts in neutrophil adhesion or extravasation into sites of inflammation or infection, and neutrophils are unable to function properly and have reduced survival if its expression is reduced or inactivated [48, 125]. Given that lisinopril and ramipril treatment showed decreased neutrophil bactericidal ability, it is postulated that ACE inhibition reduces their antimicrobial impact through reduced LTB_4_ production [48]. ACE expression also altered LTB_4_ production and could be ameliorated with LTB_4_ inhibition, resulting in reduced MRSA clearance in transgenic mice and human neutrophils [48]. LTA_4_ hydrolase metabolizes LTA_4_ into LTB_4_ through the 5-lipoxygenase pathway [126, 127]. This conversion could be inhibited through captopril, but only LTA_4_ hydrolase activity and not 5-lipoxygenase [124, 127]. In contrast, ARB administration had no impact on the bactericidal ability of neutrophils and could serve as an alternative treatment in patients at risk of infection in certain settings.

In mouse models, ACE inhibitors showed no inherent antimicrobial activity, but treatment resulted in decreased leukocyte infiltration and ROS production in a spinal implant model challenged with *Staphylococcus aureus* [125]. *In vivo* and *in vitro* murine studies had improved bacterial clearance after *L. monocytogenes* and MRSA infection when ACE was overexpressed, but when ACE was deficient, mice experienced lethal infections which could be due to decreased clearance upon ACE inhibition. Thus, it may take longer for an infection to clear if ACE is sufficiently inhibited in innate immune cells [41]. The mechanisms responsible may include reduced iNOS, eNOS, ROS, and phagocytic ability linked to ACE inhibition [41, 44, 48].

Captopril may either enhance or inhibit peptide processing and presentation depending on peptide length. Antigen presentation is enhanced for short peptides, but processing may be inhibited for longer peptides. In murine macrophages, antigen presentation is augmented with captopril treatment in conjunction with diuretic medication, leading to improved surface marker expression and phagocytosis [128]. However, this combination also reduces their ability to produce proinflammatory cytokines and upregulates IL-10, an immunomodulatory cytokine [128]. These macrophages may thus favour the M2 phenotype with improved phagocytic ability, allowing efficient bacterial clearance but impaired immune signalling or honing [129]. Ang II was initially thought to enhance phagocytosis, but this work was subsequently challenged by studies showing that Ang II had no effect, whilst ACE overexpression enhanced phagocytic ability [44, 64]. The conflicting literature is a reflection of technological advancements and changing dose-dependent treatment strategies over time [64]. ARBs are also unable to impact phagocytic ability in ACE 10/10 murine macrophages, where ACE inhibition decreases phagocytic efficiency to wild-type or lower levels [44].

During contact hypersensitivity reactions, captopril and diuretics modulate the murine macrophage cellular immune response through increased reactive oxygen intermediates but decreased NO and IL-12 secretion [130]. These effects do not influence the antimicrobial immune responses in macrophages, unlike in neutrophils treated with lisinopril and ramipril, which may support effective treatment of hypertension and contact hypersensitivity without increased risk of infection [48, 130].

#### 3.3.3. Viral Infections

In 2019, the novel coronavirus SARS-CoV2 was identified as a concerning pathogen in Wuhan, China [131, 132]. As of 2022, the SARS-CoV-2 pandemic remains a global concern, with the emergence of new variants and waves driving infections throughout the world. As the receptor for cell entry and infection was identified as ACE2, there has been much debate surrounding the safety of ACE inhibitors during infection. Currently, there is no evidence that continuing ACEi or ARB treatment presents a higher risk of severe symptoms during SARS-CoV2 infection [131, 133]. However, given the potential role of ACE in neutrophil activation and adhesion, it is worth noting that its inhibition during viral infections may have some impact on outcomes [48].

Neutrophils are crucial during viral infections as immune first responders, and during severe respiratory disease, neutrophil infiltration increases in the lungs where chromatin and granules are expelled to create neutrophil extracellular traps (NETs) that aid in halting pathogen spread. Whilst they are more prominent in bacterial infections, NETs promote immunothrombosis in SARS-CoV2 patients, indicating severe lung stress and disease [134]. Patients with severe SARS-CoV2 experience a dysregulated proinflammatory response that triggers enhanced neutrophil extravasation into the lungs and heart, where degranulation and NET formation damage host and infected cells. IL-8, a chemoattractant for neutrophils, is upregulated during SARS-CoV2 infection and therefore drives the harmful neutrophilic profile in severe cases. IL-8 expression is influenced by Ang II production, and an increase in Ang II causes an increase in IL-8 [15, 134]. In addition, THP-1 macrophage exposure to spike protein has increased MCP-1, ROS, intracellular calcium release, and apoptosis [135]. Following combination spike protein and perindopril treatment, ROS, apoptosis, and MCP-1 are significantly decreased with only a slight decline in calcium release [135]. In PBMCs, joint perindopril and spike protein treatment have decreased TNF-*α* expression and decreased IL-17 and TNF-*α* in CD4^+^ T cells; however, no effect on ROS generation is observed [135].

Although SARS-CoV2 does not directly interact with ACE or Ang II, its binding to ACE2 allows Ang II to favour AT_1_R interaction and induce a proinflammatory response [131, 132, 134]. Hypertensive patients are at increased risk of severe SARS-CoV2 infection, but other factors contribute to treatment success including age and gender. These confounding variables must be corrected for when investigating the impact of ACEi and ARBs in future COVID-19 studies. There is currently conflicting evidence on the safety of ACEi and ARB therapies in patients with COVID-19. Some studies have shown an increase in Ang II levels, linked to severe disease with reduced ACE2 levels, whilst others indicated that ACEi and ARBs may increase ACE2 levels and facilitate a higher risk of infection [131]. However, there is a lack of evidence for the latter, and most studies have concluded that antihypertensive medications may improve and protect against Ang II-induced fibrosis [136–138]. Thus, ACEi and ARB treatments should continue during COVID-19 infection. ARB inhibition may be preferable to ACE inhibition to account for reduced neutrophil and macrophage abilities as hypothesized by Cao et al., but this remains hypothetical [48].

## 4. Conclusion

Research relating to ACE inhibition and immune impact is limited to cytokine production, adaptive immunity, and autoimmune diseases with conflicting evidence across multiple studies regarding immune advantage or disadvantage [78]. ACE and Ang II have increasing roles in mediating inflammation and altering immune cell populations as presented above. In autoimmunity, ACE may be a candidate marker or gene to determine if patients will have severe cases of SLE and RA, whilst its inhibition has shown promise in maintaining inflammation and associated injury in lupus patients. In contrast, ARB prescription is favoured in human IBD studies to improve patient conditions over ACEi treatment. Additionally, cancer therapies may benefit from using ACEi in controlling chemotherapy side effects or preventing tumour progression entirely, but this appears to be cancer-dependent. Detailed work investigating the function of these inhibitors on innate immune cell populations would be beneficial in both realizing if patients require treatment adjustments in postsurgical situations where bacterial or viral infections are common or if they can be used as immunotherapeutics in replacement or conjunction with steroids and more common treatment plans. To date, large-scale data does not suggest those on ACEi are more at risk than those on ARBs, but emerging ACE overexpression and WT work indicate a more important role of intrinsic ACE in neutrophil and macrophage function than previously thought. Since ACE is a promiscuous enzyme with several substrates and biological effects, it follows that ACEi need to be specifically designed to offset unwanted side effects and enable the maintenance of homeostasis. This is particularly true in immune pathways, as our understanding of how ACE and ACE inhibitor function is expected to play an important role in the prevention and control of disease.
